# Nutritional habits according to age and BMI of 6–17-year-old children from the urban municipality in Poland

**DOI:** 10.1186/s41043-022-00296-9

**Published:** 2022-05-07

**Authors:** Alicja Basiak-Rasała, Sara Górna, Joanna Krajewska, Mateusz Kolator, Katarzyna Pazdro-Zastawny, Aleksander Basiak, Tomasz Zatoński

**Affiliations:** 1 Department of Population Health, Uniwersytet Medyczny im. Piastów Śląskich We Wrocławiu, ul. Bujwida 44, 50-345 Wrocław, Poland; 2 Department of Physiology and Biochemistry, Poznań University of Physical Education, Poznań, Poland; 3 Department of Otolaryngology, Head and Neck Surgery, Uniwersytet Medyczny im. Piastów Śląskich We Wrocławiu, Borowska 213, 50-556 Wrocław, Poland; 4 Department of Paediatric Endocrinology and Diabetology, Uniwersytet Medyczny im. Piastów Śląskich We Wrocławiu, ul. T. Chałubińskiego 2a, 50-568 Wrocław, Poland; 5 „Biegaj Dla Zdrowia” Foundation, ul. Cesarzowicka 38, 52-408 Wrocław, Poland

**Keywords:** Children, Obesity, Nutritional habits

## Abstract

**Background:**

Balanced nutrition is crucial for adolescent’s proper physical and mental development. Dietary habits change significantly with a child’s development. Along with increasing age and the shift towards adolescence, unhealthy diet-related habits become more common. The objective of the survey study was to determine the differences in nutritional habits between children and adolescents according to their age and body mass index (BMI).

**Methods:**

“Let’s get the kids moving” campaign (pol. “Uruchamiamy dzieciaki”) was launched in 2016. Within the campaign, the survey study was conducted in 2913 participants between 6 and 17 years old from primary and junior high schools in Wroclaw (Poland). The survey was anonymous, and its supplement was voluntary. Participants were divided into age groups. The study group of 2913 consisted of 29.8% of 6–9-year-olds, 32.7% of 10–12-year-olds, and 37.5% of 13–17-year-olds. Body mass index (BMI) was calculated and further interpreted as a BMI z-scores depending on children’s age and gender.

**Results:**

A total of 19.3% of participants consumed 3 meals a day or less. Children from the oldest age group (13–17) consumed statistically significantly fewer meals per day than younger children (*p* < 0.001). Children from the oldest age group (13–17) consumed breakfast statistically less often than children of age group 10–12 years (75.0% vs. 83.6%; *p* < 0.001) and children of age group 6–9 years (75.0% vs. 84.0%; *p* < 0.001). Severely thin children consumed breakfast significantly more often than overweight (85.8% vs. 76.3%; *p* = 0.004) and children with obesity (85.8% vs. 75.9%; *p* = 0.021). Children with obesity consumed vegetables significantly less often than severely thin (*p* < 0.008), thin (*p* < 0.001), and children with normal body weight (*p* < 0.007). The oldest children (13–17 years) consumed Coca-Cola and SSB (*p* < 0.001) and fruit-flavored beverages (*p* < 0.05) significantly more often than children from other age groups. Boys consumed carbonated beverages with added sugar significantly more often than girls (*p* < 0.01).

**Conclusions:**

Unhealthy diet-related behaviors in children and adolescents may promote overweight and obesity and should be targeted in health promotion programs. Special attention should be paid to 13–17-year-olds, as adolescents from this group made more unhealthy choices than younger children.

## Introduction

Balanced nutrition is crucial for adolescent’s proper physical and mental development. Deterioration of nutritional status, both in case of excessive or insufficient intake of macro- and micronutrients, can result in severe health consequences. Nowadays, the prevalence of overweight and obesity is alarmingly increasing in children and adolescents, increasing the risk of noncommunicable diseases in adulthood and, as a result, premature death [1]. It is estimated that 57.3% of children with obesity between the age of 2 and 19 in 2016 will continue to be obese at the age of 35 years [2]. Moreover, approximately 81% of adolescents globally do not achieve the recommended 60 min of physical activity per day [3]. Overweight and obesity result from exposure to the composition of genetic, environmental, biological, and behavioral risk factors [4, 5]. It is believed that genetic factors are responsible for less than 5% of cases of childhood obesity [6]. The high prevalence of overweight and obesity can be attributed mostly to modifiable risk factors. Both low physical activity and improper nutrition can contribute to excess body weight. According to data from Health Behavior in School-Aged Children Study (HBSC) conducted in 2009–2010, 36% of boys and 23% of girls among 11-year-olds; 28% of boys and 16% of girls among 13-year-olds; and 20% of boys and 12% of girls among 15-year-olds in Poland were overweight [7]. According to observations in the HBSC study in Poland, the prevalence of obesity in 11–15 year-olds increased significantly over time from 2002 to 2014 in both genders [8]. Recently published polish nutritional guidelines for children and adolescents changed the recommendations regarding consumption of some of the food groups [9]. Major change concerned vegetables and fruit, which were placed at the base of the nutritional pyramid, below whole-grain cereals, which were initially there, which means that vegetables and fruit should be consumed in the largest amount and added to every meal. Developing healthy eating habits is one of the many preventive measures that can be undertaken to decrease the risk of the development of noncommunicable diseases in adulthood.

Considering the aforementioned alarming tendencies, children and adolescents are candidates for primary prevention programs, hence, launching the “Let’s get the kids moving” campaign (pol. “Uruchamiamy dzieciaki”). Initially, the campaign targeted physical education teachers, parents, and coaches to increase their knowledge on the prevalence of overweight, overweight-related health problems, balanced nutrition, and prevention of obesity among adolescents. The program aimed also to conduct a survey study to diagnose nutritional habits, level of physical fitness, leisure time activities, general health status, and condition of the musculoskeletal system of students from primary and junior high schools in Wroclaw (Poland). There are several physical activity-oriented campaigns currently conducted in Poland. The programs such as “Athletics for all” [10], “School sports club” [11], “Keep fit!” [12] aimed to provide regular athletics training in children and adolescents in Poland. Although improving awareness about the basics of healthy nutrition in participants was usually a secondary aim in such projects, the nutritional habits of participants were rarely investigated within the programs [12]. To date, several local and nationwide studies conducted in Poland focused on nutritional habits and frequency of consumption of food groups in children and adolescents [7, 13–21].

The objective of the study was the assessment of nutritional habits of children and adolescents from primary and junior high schools in Wroclaw, Poland, and to determine whether the correlation exists between the nutritional habits in children according to their age and body mass index (BMI).

## Methods

“Let’s get the kids moving” campaign (pol. “Uruchamiamy dzieciaki”) was launched in 2016 and created and organized by the employees of Wroclaw Medical University, University Clinical Hospital in Wroclaw and “Run for Health” Foundation (pol. “Biegaj dla Zdrowia”). Wrocław is a large city of 640 000 inhabitants in Lower Silesia, Poland. The project design was described in detail by Pazdro-Zastawny et al. [22]. The main aim of the overall project was to increase physical activity and prevent overweight and obesity in children and adolescents. A total of 34 primary and junior high schools in Wrocław were recruited. The campaign was primarily addressed to teachers, parents, and trainers to support school environments and families by improving the knowledge of a healthy lifestyle. A series of lectures on healthy lifestyle and health-related topics were organized. The project included also conducting 12-min Cooper’s test for children in every participating school [22]. Additionally, a survey study was conducted. Questionnaires were distributed to primary and junior high schools in Wroclaw and handed out to parents/guardians during periodic school meetings. We observed a response rate of 29%. A total of 3344 questionnaires completed by parents/guardians of children aged between 6 and 17 years were returned. We excluded 431 surveys from hereby analysis due to incomplete data. A total of 2913 complete surveys were analyzed.

The original questionnaire used was developed especially for this study, but it was not validated. The survey was anonymous, and its supplement was voluntary. The questionnaire consisted of three sections regarding 1) physical activity; 2) nutritional habits; and 3) health status. Questionnaires were distributed to schools’ coordinators and handed out to parents during periodic school meetings. Hereby manuscript analyzed the data from the second part of the questionnaire concerning nutritional habits.

Part of the survey regarding nutritional habits consisted of 8 questions, concerning the number of meals, consumption of snacks and first breakfast, consumption of meals outside home or school, and following any specific diet (e.g., gluten-free, reducing diet). Moreover, participants were asked to assess how often were they consuming 18 following groups of products: (1) vegetables; (2) fruit; (3) potatoes; (4) milk and dairy products; (5) whole-grain cereal products, like rye bread or groats; (6) refined-grain cereal products, like wheat bread and white rice; (7) fish; (8) poultry; (9) red meat; (10) charcuterie including sausages; (11) eggs; (12) sweets; (13) salty snacks including crisps; (14) Coca-Cola or other sugar-sweetened beverages; (15) fruit juices; (16) fruit-flavored non-carbonated beverages including flavored mineral waters; (17) mineral water; (18) fast-food dishes. Participants could have chosen the following frequencies: (1) never; (2) 1–2 times a month; (3) 1–2 times a week; (4) 3–4 times a week; (5) 5–6 times a week; (6) every day, once a day; (7) every day, several times a day.

For analysis, participants were divided into three age groups: 6–9 years old, 10–12 years old, and 13–17 years old. Based on the weight and height of participants reported by their parents/legal guardians, body mass index (BMI) was calculated and further interpreted as BMI z-scores depending on children’s age and gender. We have interpreted cutoffs in z-scores according to the World Health Organization (WHO) recommendations (obesity > + 2SD; overweight > + 1SD < ; -2SD ≤ normal body weight ≤ + 1; thinness < -2SD > ; severe thinness < -2SD) [23, 24].

Statistical analysis was performed using the Statistica software version 13.1. PL (StatSoft, Inc., USA). Normal distribution was verified by the Kolmogorov–Smirnov test. The results between the groups were compared using nonparametric Kruskal–Wallis test and Chi-squared test. For all analyses, the statistical significance was set at *p* = 0.05. The study was approved by the Wroclaw Medical University Ethics Committee (No. 738/2018).

## Results

A total of 2913 eligible surveys were included in the analysis. The study group consisted of 48.7% girls and 51.3% boys. The survey was completed mostly by mothers (82.6%). The majority (92.3%) of participants lived in Wroclaw. The study population consisted of 29.8% of 6–9 year-olds, 32.7% of 10–12 year-olds, and 37.5% of 13–17-year-olds. According to BMI, 9% of the population was severely thin, 18% was thin, 58% had normal body weight, 11% was overweight, and 4% was obese. Basic characteristics of the study population are presented in Table 1.

**Table 1:** Sociodemographic characteristic of the study population (*n* = 2913) according to the age groups

	Overall *n* (%)	Age group			*p*
		6–9 years *n* (%)	10–12 years *n* (%)	13–17 years *n* (%)	
*Sex*					
Boys	1419 (48.7)	413 (47.6)	456 (47.8)	550 (50.4)	0.384
Girls	1494 (51.3)	454 (52.4)	498 (52.2)	542 (49.6)	
*Place of residence*					
Rural	201 (6.9)	48 (5.5)	66 (6.9)	87 (8.0)	0.067
Small city (< 20 000 citizens)	16 (0.5)	4 (0.5)	3 (0.3)	9 (0.8)	
Average city (20 000–100 000 citizens)	8 (0.3)	0 (0.0)	3 (0.3)	5 (0.5)	
Big city (Wroclaw) (> 100 000 citizens)	2688 (92.3)	815 (94.0)	882 (92.4)	991 (90.8)	
*BMI (kg/m*^*2*^*)*					
M ± SD	18.1 ± 3.3	16.1 ± 2.5	17.7 ± 3.1	19.9 ± 3.1	< 0.001*
*Me* [*Q*_1_; *Q*_3_]	18 [16; 20]	16 [14; 17]	17 [16; 19]	20 [18; 21]	
*Min*—*Max*	9–40	10–32	11–40	9–33	

Statistically significant differences between groups (*p* ≤ 0.05) are indicated by *

BMI, body mass index; M, mean; SD, standard deviation; Me, median; Q_1_, lower quartile; Q_3_, upper quartile; Min, minimum value; Max, maximum value; *—statistically significant result

A total of 19.3% of participants consumed 3 meals a day or less. The majority (74.5%) of participants declared consumption of five meals a day. Children from the oldest age group (13–17) consumed statistically significantly fewer meals per day than younger children (*p* < 0.001). There were no statistical differences between genders and BMI regarding the number of meals per day. A sum of 66% of participants consumed snacks between meals, regardless of sex, age, and BMI (*p* > 0.05). A total of 80.5% of children consumed breakfast before going to school. There were statistically significant differences between age groups and BMI. Children from the oldest age group (13–17) consumed breakfast statistically less often than children of age group 10–12 years (75.0% vs. 83.6%; *p* < 0.001) and children of age group 6–9 years (75.0% vs. 84.0%; *p* < 0.001). Severely thin children consumed breakfast significantly more often than overweight children (85.8% vs. 76.3%; *p* = 0.004) and children with obesity (85.8% vs. 75.9%; *p* = 0.021). There were significant differences between the consumption of additional meals outside the home or school. Children of the oldest age group consumed such meals significantly more often than the youngest children (37.5% vs. 32.2%; *p* < 0.017). Thin children consumed additional meals significantly more often than children with normal body weight (39.8% vs. 33.6%; *p* = 0.008) and overweight children (39.8% vs. 30.3%; *p* = 0.002). There were no significant differences regarding the consumption of additional meals between genders.

Results of the frequency of consumption of food groups in the whole population are presented in Table 2. There were significant differences between the consumption of some of the food groups. One-fourth (24.6%) of all children consumed vegetables several times a day. The youngest children (6–9 years) consumed vegetables significantly more often than the oldest children (13–17 years) (*p* < 0.001). Children with obesity according to BMI consumed vegetables significantly less often than severely thin (*p* < 0.008), thin (*p* < 0.001), and children with normal body weight (*p* < 0.007). A total of 27.2% of all children consumed fruit several times a day. The youngest children (6–9 years) consumed fruit significantly more often than the older (10–12 years) and the oldest children (13–17 years) (*p* < 0.01). Older children (10–12 years) consumed significantly more fruit than the oldest children (13–17 years) (*p* < 0.001). Girls consumed fruit significantly more often than boys (*p* < 0.05).

**Table 2:** Frequency of consumption of 18 food groups included in a questionnaire reported in a study populations (2913 participants)

	Never	1–2 times a month	1–2 times a week	3–4 times a week	5–6 times a week	Everyday, 1 time a day	Everyday, several times a day
Vegetables *n (%)*	25 (0.9)	74 (2.5)	330 (11.3)	477 (16.4)	390 (13.4)	900 (30.9)	717 (24.6)
Fruit *n (%)*	18 (0.6)	34 (1.2)	238 (8.2)	477 (16.4)	404 (13.9)	951 (32.6)	791 (27.2)
Potatoes *n (%)*	28 (1.0)	183 (6.3)	860 (29.5)	1172 (40.2)	373 (12.8)	246 (8.4)	51 (1.8)
Milk and dairy products *n (%)*	65 (2.2)	78 (2.7)	250 (8.6)	501 (17.2)	453 (15.6)	959 (32.9)	607 (20.8)
Whole-grain cereal products, like rye bread and groats *n (%)*	164 (5.6)	395 (13.6)	717 (24.6)	591 (20.3)	309 (10.6)	452 (15.5)	285 (9.8)
Refined-grain cereal products, like wheat bread, white rice *n (%)*	67 (2.3)	239 (8.2)	609 (20.9)	691 (23.7)	413 (14.2)	619 (21.2)	275 (9.4)
Fish *n (%)*	273 (9.4)	1116 (38.3)	1316 (45.2)	163 (5.6)	24 (0.8)	17 (0.6)	4 (0.1)
Poultry (chicken, turkey) *n (%)*	68 (2.3)	171 (5.9)	1258 (43.2)	1087 (37.3)	245 (8.4)	73 (2.5)	11 (0.4)
Red meat (pork, beef) *n (%)*	250 (8.6)	887 (30.4)	1334 (45.8)	358 (12.3)	53 (1.8)	20 (0.7)	11 (0.4)
Charcuterie (sausages, ham, pate) *n (%)*	162 (5.6)	322 (11.1)	804 (27.6)	770 (26.4)	431 (14.8)	334 (11.5)	90 (3.1)
Eggs *n (%)*	110 (3.8)	293 (10.1)	1427 (49.0)	754 (25.9)	233 (8.0)	76 (2.6)	20 (0.7)
Sweets *n (%)*	35 (1.2)	184 (6.3)	715 (24.5)	736 (25.3)	429 (14.7)	622 (21.4)	192 (6.6)
Salty snacks *n (%)*	317 (10.9)	1312 (45.0)	819 (28.1)	290 (10.0)	80 (2.7)	67 (2.3)	28 (1.0)
Coca-Cola and sugar-sweetened beverages *n (%)*	1008 (34.6)	1232 (42.3)	441 (15.1)	129 (4.4)	43 (1.5)	37 (1.3)	23 (0.8)
Fruit juices *n (%)*	99 (3.4)	524 (18.0)	742 (25.5)	657 (22.6)	365 (12.5)	330 (11.3)	196 (6.7)
Fruit drinks including flavored mineral waters *n (%)*	716 (24.6)	804 (27.6)	516 (17.7)	337 (11.6)	184 (6.3)	212 (7.3)	144 (4.9)
Mineral water *n (%)*	29 (0.1)	64 (2.2)	140 (4.8)	155 (5.3)	201 (6.9)	529 (18.2)	1795 (61.6)
Fast-food *n (%)*	380 (13.0)	2125 (72.9)	339 (11.6)	47 (1.6)	10 (0.3)	8 (0.3)	4 (0.1)

Almost one-third (32.9%) of participants consumed milk and dairy products once a day, whereas 20.8% consumed such products several times a day. There were no significant differences in consumption of milk and dairy products between sexes, age groups, and BMI. Almost half (45.2%) of participants declared that children consume fish 1–2 times a week. The youngest children (6–9 years) consumed fish significantly more often than other children (*p* < 0.05). Children consumed poultry more often than red meat. A total of 43.2% of participants consumed poultry 1–2 times a week, 37.3% 3–4 times a week, and 8.4% 5–6 times a week. Majority of participants (45.8%) consumed red meat 1–2 times a week, 30.4% 1–2 times a month. Charcuterie was a food group that also occurred frequently in children’s diets. A little over a quarter (27.6%) of participants consumed charcuterie 1–2 times a week, 26.4% 3–4 times a week, 14.8% 5–6 times a week, 11.5% once a day. There were no significant differences in the consumption of poultry, red meat, and charcuterie between genders, BMI, and age groups.

Most participants (45.0%) declared consumption of salty snacks 1–2 times a month, 28.1% 1–2 times a week. The oldest children (13–17 years) consumed salty snacks (*p* < 0.001), significantly more often than children from other age groups. Children with obesity consumed salty snacks significantly more often than thin children (*p* < 0.036).

The majority of participants (42.3%) consumed Coca-Cola and sugar-sweetened beverages (SSB) 1–2 times a month. The oldest children (13–17 years) consumed Coca-Cola and SSB (*p* < 0.001) and fruit-flavored beverages (*p* < 0.05) significantly more often than children from other age groups. Boys consumed carbonated beverages with added sugar significantly more often than girls (*p* < 0.01). Severely thin children consumed Coca-Cola and SSB significantly more often than overweight (*p* = 0.022) and children with obesity (*p* = 0.022). Thin children consumed Coca-Cola and SSB and fruit-flavored beverages significantly more often than overweight children (*p* = 0.047 and *p* < 0.027, respectively). Girls drank mineral water significantly more often than boys (*p* < 0.05).

Majority of participants (72.9%) consumed fast-food products 1–2 times a month. Boys consumed fast-food meals significantly more often than girls (*p* < 0.05). The oldest children (13–17 years) consumed fast-food products significantly more often than children from other age groups (*p* < 0.001).

No statistical differences between genders, age groups, or BMI were observed in the frequency of consumption of the following food groups: potatoes, whole-grain, and refined cereal products, eggs, sweets, fruit juices. The frequency of consumption of food groups according to the age of the participants is presented in Table 3.

**Table 3:** Frequency of consumption of 18 food groups differentiated by age groups of the participants

Food group	Age groups							p**
	6–9 years *N* = 867							
	Never	1–2 times a month	1–2 times a week	3–4 times a week	5–6 times a week	Everyday, once a day	Everyday, several times a day	
	*n* (%)							
Vegetables	4 (0.5)	19 (2.2)	88 (10.1)	134 (15.5)	98 (11.3)	286 (33.0)	238 (27.5)	0.001*
Fruit	3 (0.3)	7 (0.8)	56 (6.5)	99 (11.4)	109 (12.6)	318 (36.7)	275 (31.7)	< 0.001*
Potatoes	8 (0.9)	38 (4.4)	250 (28.8)	384 (44.3)	99 (11.4)	73 (8.4)	15 (1.7)	0.454
Milk and dairy products	18 (2.1)	25 (2.9)	70 (8.1)	144 (16.6)	125 (14.4)	282 (32.5)	203 (23.4)	0.113
Whole-grain cereals	49 (5.7)	89 (10.3)	246 (28.4)	183 (21.1)	91 (10.5)	129 (14.9)	80 (9.2)	0.289
Refined-grain cereal products	11 (1.3)	61 (7.0)	188 (21.7)	221 (25.5)	105 (12.1)	195 (22.5)	86 (9.9)	0.450
Fish	65 (7.5)	315 (36.3)	429 (49.5)	47 (5.4)	7 (0.8)	3 (0.3)	1 (0.1)	0.019*
Poultry	12 (1.4)	48 (5.5)	403 (46.5)	312 (36.0)	67 (7.7)	21 (2.4)	4 (0.5)	0.653
Red meat	57 (6.6)	256 (29.5)	433 (49.9)	93 (10.7)	17 (2.0)	8 (0.9)	3 (0.3)	0.072
Charcuterie	27 (3.1)	83 (9.6)	264 (30.4)	245 (28.3)	123 (14.2)	97 (11.2)	28 (3.2)	0.202
Eggs	34 (3.9)	81 (9.3)	445 (51.3)	223 (25.7)	67 (7.7)	14 (1.6)	3 (0.3)	0.533
Sweets	3 (0.3)	35 (4.0)	252 (29.1)	235 (27.1)	125 (14.4)	182 (21.0)	35 (4.0)	0.109
Salty snacks	122 (14.1)	439 (50.6)	228 (26.3)	48 (5.5)	15 (1.7)	11 (1.3)	4 (0.5)	< 0.001*
Coca-Cola and SSB	454 (52.4)	316 (36.4)	79 (9.1)	8 (0.9)	6 (0.7)	2 (0.2)	2 (0.2)	< 0.001*
Fruit juices	23 (2.7)	164 (18.9)	222 (25.6)	196 (22.6)	100 (11.5)	96 (11.1)	66 (7.6)	0.383
Fruit-flavored drinks	244 (28.1)	236 (27.2)	152 (17.5)	88 (10.1)	51 (5.9)	56 (6.5)	40 (4.6)	< 0.001*
Mineral water	6 (0.7)	13 (1.5)	44 (5.1)	47 (5.4)	53 (6.1)	162 (18.7)	542 (62.5)	0.431
Fast-food	134 (15.5)	659 (76.0)	64 (7.4)	4 (0.5)	5 (0.6)	0 (0.0)	1 (0.1)	< 0.001*

Food group	Age groups							p**
	10–12 years *N* = 954							
	Never	1–2 times a month	1–2 times a week	3–4 times a week	5–6 times a week	Everyday, once a day	Everyday, several times a day	
	*n* (%)							
Vegetables	10 (1.0)	16 (1.7)	109 (11.4)	160 (16.8)	143 (15.0)	277 (29.0)	239 (25.1)	0.001*
Fruit	8 (0.8)	12 (1.3)	71 (7.4)	148 (15.5)	145 (15.2)	306 (32.1)	264 (27.7)	< 0.001*
Potatoes	4 (0.4)	63 (6.6)	280 (29.4)	388 (40.7)	137 (14.4)	69 (7.2)	13 (1.4)	0.454
Milk and dairy products	22 (2.3)	19 (2.0)	89 (9.3)	162 (17.0)	149 (15.6)	313 (32.8)	200 (21.0)	0.113
Whole-grain cereals	59 (6.2)	139 (14.6)	226 (23.7)	201 (21.1)	110 (11.5)	131 (13.7)	88 (9.2)	0.289
Refined-grain cereal products	15 (1.6)	74 (7.8)	201 (21.1)	238 (24.9)	153 (16.0)	202 (21.2)	71 (7.4)	0.450
Fish	89 (9.3)	371 (38.9)	420 (44.0)	62 (6.5)	7 (0.7)	3 (0.3)	2 (0.2)	0.019*
Poultry	12 (1.3)	58 (6.1)	412 (43.2)	364 (38.2)	90 (9.4)	17 (1.8)	1 (0.1)	0.653
Red meat	72 (7.5)	305 (32.0)	413 (43.3)	138 (14.5)	20 (2.1)	3 (0.3)	3 (0.3)	0.072
Charcuterie	39 (4.1)	119 (12.5)	250 (26.2)	267 (28.0)	152 (15.9)	101 (10.6)	26 (2.7)	0.202
Eggs	43 (4.5)	97 (10.2)	441 (46.2)	264 (27.7)	78 (8.2)	26 (2.7)	5 (0.5)	0.533
Sweets	10 (1.0)	64 (6.7)	214 (22.4)	232 (24.3)	148 (15.5)	220 (23.1)	66 (6.9)	0.109
Salty snacks	107 (11.2)	438 (45.9)	256 (26.8)	100 (10.5)	29 (3.0)	19 (2.0)	5 (0.5)	< 0.001*
Coca-Cola and SSB	303 (31.8)	474 (49.7)	127 (13.3)	36 (3.8)	5 (0.5)	5 (0.5)	4 (0.4)	< 0.001*
Fruit juices	27 (2.8)	183 (19.2)	262 (27.5)	198 (20.8)	113 (11.8)	116 (12.2)	55 (5.8)	0.383
Fruit-flavored drinks	237 (24.8)	280 (29.4)	163 (17.1)	98 (10.3)	60 (6.3)	76 (8.0)	40 (4.2)	< 0.001*
Mineral water	8 (0.8)	24 (2.5)	44 (4.6)	42 (4.4)	65 (6.8)	181 (19.0)	590 (61.8)	0.431
Fast-food	123 (12.9)	718 (75.3)	97 (10.2)	10 (1.0)	2 (0.2)	3 (0.3)	1 (0.1)	< 0.001*

Food group	Age groups							p**
	13–17 years *N* = 1092							
	Never	1–2 times a month	1–2 times a week	3–4 times a week	5–6 times a week	Everyday, once a day	Everyday, several times a day	
	*n* (%)							
Vegetables	11 (1.0)	39 (3.6)	133 (12.2)	183 (16.8)	149 (13.6)	337 (30.9)	240 (22.0)	0.001*
Fruit	7 (0.6)	15 (1.4)	111 (10.2)	230 (21.1)	150 (13.7)	327 (29.9)	252 (23.1)	< 0.001*
Potatoes	16 (1.5)	82 (7.5)	330 (30.2)	400 (36.6)	137 (12.5)	104 (9.5)	23 (2.1)	0.454
Milk and dairy products	25 (2.3)	34 (3.1)	91 (8.3)	195 (17.9)	179 (16.4)	364 (33.3)	204 (18.7)	0.113
Whole-grain cereals	56 (5.1)	167 (15.3)	245 (22.4)	207 (19.0)	108 (9.9)	192 (17.6)	117 (10.7)	0.289
Refined-grain cereal products	41 (3.8)	104 (9.5)	220 (20.1)	232 (21.2)	155 (14.2)	222 (20.3)	118 (10.8)	0.450
Fish	119 (10.9)	430 (39.4)	467 (42.8)	54 (4.9)	10 (0.9)	11 (1.0)	1 (0.1)	0.019*
Poultry	44 (4.0)	65 (6.0)	443 (40.6)	411 (37.6)	88 (8.1)	35 (3.2)	6 (0.5)	0.653
Red meat	121 (11.1)	326 (29.9)	488 (44.7)	127 (11.6)	16 (1.5)	9 (0.8)	5 (0.5)	0.072
Charcuterie	96 (8.8)	120 (11.0)	290 (26.6)	258 (23.6)	156 (14.3)	136 (12.5)	36 (3.3)	0.202
Eggs	33 (3.0)	115 (10.5)	541 (49.5)	267 (24.5)	88 (8.1)	36 (3.3)	12 (1.1)	0.533
Sweets	22 (2.0)	85 (7.8)	249 (22.8)	269 (24.6)	156 (14.3)	220 (20.1)	91 (8.3)	0.109
Salty snacks	88 (8.1)	435 (39.8)	335 (30.7)	142 (13.0)	36 (3.3)	37 (3.4)	19 (1.7)	< 0.001*
Coca-Cola and SSB	251 (23.0)	442 (40.5)	235 (21.5)	85 (7.8)	32 (2.9)	30 (2.7)	17 (1.6)	< 0.001*
Fruit juices	49 (4.5)	177 (16.2)	258 (23.6)	263 (24.1)	152 (13.9)	118 (10.8)	75 (6.9)	0.383
Fruit-flavored drinks	235 (21.5)	288 (26.4)	201 (18.4)	151 (13.8)	73 (6.7)	80 (7.3)	64 (5.9)	< 0.001*
Mineral water	15 (1.4)	27 (2.5)	52 (4.8)	66 (6.0)	83 (7.6)	186 (17.0)	663 (60.7)	0.431
Fast-food	123 (11.3)	748 (68.5)	178 (16.3)	33 (3.0)	3 (0.3)	5 (0.5)	2 (0.2)	< 0.001*

^*^Statistically significant differences between groups (*p* ≤ 0.05); **Kruskal–Wallis test, post hoc analysis

## Discussion

The analysis revealed some unhealthy diet-related behaviors common in the whole or part of the population. Dietary guidelines [9] recommend that children and adolescents should consume meals regularly, every 3–4 h, which sums up to 5 meals a day. Almost one-fifth of the population consumed fewer meals than recommended. On the other hand, most participants declared daily consumption of snacks, mostly sweets, and fruit. Consumption of fewer meals than recommended was slightly more common (approximately 40% of the population) in a study by Dolipska et al. [15] conducted in Śląskie, Opolskie, and Małopolskie voivodeships in Poland. Although we haven’t observed that the number of consumed meals per day was associated with the BMI of the participants, some other polish studies reported that consumption of fewer than 3 meals per day was more common in overweight and obese children [18, 21].

About one-fifth of the population didn’t consume breakfasts before attending school, which was similar to the percentage observed in Poland according to Health Behavior in School-Aged Children (HBSC) Study Report [7] and local studies [13, 15]. Consumption of breakfast is considered healthy behavior especially important in developmental age as it is associated with better cognitive function later in the day [25], better overall diet quality [26], and higher intake of fiber and micronutrients [27]. Skipping breakfast is also a behavior associated with an increased risk of excessive body mass in adolescents [28], and we also observed that skipping breakfast was more prevalent in children with overweight and obesity than in severely thin children. We also observed that skipping breakfast was more prevalent in the oldest age group (13–17) than in younger children, and this tendency was also reported in HBSC Study [7] and the local polish study conducted by Wojtyła-Buciora et al. [13] and Myszkowska-Ryciak et al. [21]. It is speculated that dietary habits are associated with school performance. According to Kim et al. [29], who conducted an analysis of more than 300,000 participants aged 12–18 years old, frequent consumption of breakfasts, fruit, vegetables, and milk was associated with better school performance, whereas higher consumption of soft drinks, instant dishes, fast food, and confectionery was negatively associated with school performance.

According to dietary guidelines for children and adolescents [9], vegetables and fruit (V&F) should be consumed as often as possible, preferably to every meal, considering however higher intake of vegetables than fruit. Increased consumption of V&F is associated with a decreased risk of cardiovascular diseases [30], type 2 diabetes [31], hypertension [32], and some types of cancer [33]. Only about one-fourth of our population consumed V&F several times a day as recommended. Although the percentage of participants, who consumed the recommended amount of V&F was low, it was, in fact, higher than observed in an HBSC study in Poland, where only 13.4% of 11–12-year-olds and 10.5% of 15–16-year-olds consumed vegetables several times a day [20]. Our observations of the frequency of consumption of V&F are in line with several local polish studies reporting the frequency of consumption of food groups in children and adolescents [17, 19]. On the other hand, Dolipska et al. [15] observed a higher frequency of consumption of V&F in primary school pupils (vegetables and fruit were consumed several times a day by 38% and 55% of participants, respectively). In the PRO GREENS study [34] conducted among 11-year-olds in 10 European countries, 23.5% of participants met recommendations of consumption of V&F, which is consistent with our findings. In the same study, 53.3% of participants did not even consume vegetables daily (in comparison 45.9% among 10–12-year-olds in our study). In our findings, the youngest children consumed V&F significantly more often than the oldest children, which is consistent with findings from the HBSC study [20]. In our study, children with obesity consumed significantly fewer vegetables than other children. Similarly, an inverse association between the consumption of vegetables and BMI was observed in the ISAAC study, which analyzed data from more than 200,000 adolescents from 36 countries [35]. Likewise, in a local polish study published by Zadka et al. [19] the frequency of V&F consumption decreased with increasing BMI of studied children and adolescents.

One-fifth of the population consumed milk and dairy products several times a day. According to dietary guidelines [9], children and adolescents should consume 3–4 servings of milk or other natural dairy products, like yogurt, buttermilk, kefir, or cheese daily, to deliver the recommended daily intake (RDI) of calcium. Milk and dairy are a rich source of high-quality protein, calcium, phosphorus, zinc, magnesium, vitamin D, and K, all of which play a crucial role in bone formation and skeletal development in childhood and adolescence [36]. Moreover, according to a meta-analysis recently conducted by Lu et al. [37], children with the highest intake of dairy were 38% less likely to have overweight and obesity in comparison with children characterized by the lowest intake. In a local polish study by Zadka et al. [19] more than half of mothers reported that children consumed dairy products once a day. In a study by Dolipska et al. [15] milk, natural yogurt, cottage cheese, and hard cheese were consumed several times a day by 20%, 11%, 7%, and 16% of primary school pupils, respectively. Conforming to our results, Stefańska et al. [14] reported that recommended amounts of milk were consumed only by 30% of 11–12-year-olds and 20% of 13–15-year-olds.

Processed meat products, like ham, sausages, paté occurred more often in the diet of participants on the daily basis (from 5 to 6 times a week to several times a day) than unprocessed poultry and red meat. Meat is an important source of high-quality protein, zinc, iron, and vitamins of the B-group. However, according to dietary guidelines, processed meat products should be avoided [9], due to higher than unprocessed meat content of saturated fatty acids, salt, nitrates, and polycyclic aromatic hydrocarbons. Increased intake of processed meat products is associated with an increased incidence of diabetes [37], cardiovascular diseases [38] and colorectal cancer [39].

Most participants consumed Coca-Cola and SSB 1–3 times a month; on the other hand, beverages like fruit juices and fruit drinks including flavored mineral waters were consumed more often. In our results, a minimal percentage of participants declared daily consumption of Coca-Cola and SSB, in contrast to results obtained by Szczepańska et al. [40], in which 37.8% of participants with normal body weight and 34.4% of overweight participants consumed SSB at least once a day. However, in the study by Szczepańska et al., there was no information on whether fruit drinks were also included in this percentage. Historically fruit juice was recommended for children as a replacement for some servings of whole fruit during the day. Up-to-date guidelines for infants, children, and adolescents by the American Academy of Pediatrics emphasize that fruit juice provides no advantage over whole fruit, but contains less fiber and more sugar, and therefore, it should be excluded from the diet of infants before 12 months of age and limited in the diet of children and teenagers between 7 and 18 years of age, as fruit juices play no essential role in a healthy, balanced diet [41]. Fruit drinks, on the other hand, are often mistakenly perceived by consumers as a product identical to fruit juice, when in fact they contain a minimum of 25% of fruit juice and often additionally added sugar, sweetener, or aroma [42]. Trying to follow nutritional guidelines, which recommend drinking 5–6 glasses of water per day [9], parents often provide children flavored mineral waters, which are more accepted by children due to their sweet taste. Flavored mineral waters are products more similar to SSB than natural water—they are waters with the addition of sugar, flavor, aroma, colorant, and preservative, but they are advertised as a replacement for natural mineral water, which can mislead consumers.

Almost one-third of the participants consumed sweets at least once a day and a similar percentage was observed in the HBSC study [20]. According to our results, sweets were among the most commonly consumed snacks between meals. In the study by Szczepańska et al. [40], 60.6% of participants with normal body weight and 54.2% of participants with overweight consumed sweets at least once a day. In more up-to-date studies [43], conducted in participants < 18 years old, daily consumption of sweets was reported in 30% of participants, which is consistent with our findings.

In our study, three-quarters of participants consumed fast-food dishes 1–3 times a month, the next 11.6% consumed such products 1–2 times a week. A higher prevalence of fast-food consumption was observed in the study by Mendyk et al. [43], where 61.1% of participants consumed fast-food products 1–3 times a month, whereas 33.6% consumed fast-food products at least once a week. On the contrary to the study by Mendyk et al. [43], in our study, individuals who completed the questionnaires were parents, which possibly, especially in the case of teenagers, could have led to some underestimation of unrecommended food groups. In a study by Dolipska et al. [15], 41% and 35% of participants reported consumption of fast-food dishes occasionally and several times a month, respectively.

In our study, children from the oldest age group (13–17 year-old) consumed breakfasts significantly less often and fewer meals per day than younger children. Moreover, they consumed additional meals outside home or school, salty snacks, Coca-Cola and SSB, fruit-flavored beverages, and fast-food dishes significantly more often than younger children and simultaneously they consumed V&F and fish significantly less often. To sum up, the oldest group of children, who received some autonomy regarding food choices, made more unhealthy diet-related choices.

There are some limitations to consider. The questionnaire used in this study was developed especially for this study, but it was not validated. Further study limitation regards parent-reporting of body weight and height of children and lack of actual measurements to confirm them. This is a common problem in studies conducted on large scale. On the other hand, according to Aasvee et al. [44], who performed validation of this method, underestimation of self-reported height and weight to anthropometric measurements in case of overweight was small and equaled 3.6%, and therefore, self-reported height and weight are still a method of choice in large survey studies. Our study presents results only from one region in Poland and they cannot be extrapolated directly to the whole Polish population. Having said that, the similarities between our results and observations from studies conducted in other regions in Poland indicate that our study accurately captures nutritional trends observed countrywide. One of the strengths to consider is a large population sample in comparison with other Polish studies.

## Conclusions

Dietary patterns in surveyed children were unsatisfactory. The participants consumed fewer meals a day than recommended and skipped breakfast before going to school. The consumption of milk and dairy products was inadequate. Revealed unhealthy diet-related behaviors in children and adolescents may promote overweight and obesity and should be targeted in health promotion programs. Special attention should be paid to 13–17-year-olds as adolescents from this group made more unhealthy choices than younger children.

## Data Availability

The datasets used and/or analyzed during the current study are available from the corresponding author on reasonable request.

## Abbreviations

HBSC: Health Behavior in School-Aged Children Study
BMI: Body mass index
V&F: Vegetables and fruit
RDI: Recommended daily intake
